# In Vitro Antitumor Activity of Endophytic and Rhizosphere Gram-Positive Bacteria from *Ibervillea sonorae* (S. Watson) Greene against L5178Y-R Lymphoma Cells

**DOI:** 10.3390/ijerph19020894

**Published:** 2022-01-14

**Authors:** Ricardo Romero-Arguelles, César Iván Romo-Sáenz, Karla Morán-Santibáñez, Patricia Tamez-Guerra, Ramiro Quintanilla-Licea, Alonso Alberto Orozco-Flores, Jesica María Ramírez-Villalobos, Reyes Tamez-Guerra, Cristina Rodríguez-Padilla, Ricardo Gomez-Flores

**Affiliations:** 1 Departamento de Microbiología e Inmunología, Facultad de Ciencias Biológicas, Universidad Autónoma de Nuevo León, San Nicolás de los Garza 66455, Mexico; ricardoromeroarguelles@gmail.com (R.R.-A.); karlita_moran@hotmail.com (K.M.-S.); patamez@hotmail.com (P.T.-G.); lacxelo@gmail.com (A.A.O.-F.); jesicamrv_901@hotmail.com (J.M.R.-V.); reyes.tamezgr@uanl.edu.mx (R.T.-G.); crrodrig07@gmail.com (C.R.-P.); 2 Departamento de Química, Universidad Autónoma de Nuevo León, San Nicolás de los Garza 66455, Mexico; rquintanilla.uanl@gmail.com

**Keywords:** Cucurbitaceae, *Ibervillea sonorae*, antitumor, *Bacillus*, *Micromonospora*, endophytic bacteria, rhizosphere, Gram-positive

## Abstract

Plant-associated microorganisms represent a potential source of new antitumor compounds. The aim of the present study was to isolate endophytic and rhizosphere Gram-positive bacteria from *Ibervillea sonorae* and produce extracts with antitumor activity. Methanol and ethyl acetate extracts were obtained from 28 d bacterial fermentation, after which murine L5178Y-R lymphoma cells growth inhibition was evaluated at concentrations ranging from 15.62 µg/mL to 500 µg/mL by the 3-[4,5-dimethylthiazol-2-yl]-2,5 diphenyl tetrazolium bromide reduction colorimetric assay. IC_50_ and the selectivity index (SI) were calculated and compared with healthy control human peripheral blood mononuclear cells (PBMC). Identification of the isolated strains was performed using the 16S ribosomal gene and by MALDI-TOF MS mass spectrometry. The endophytic and rhizosphere bacterial extracts from strains ISE-B22, ISE-B26, ISE-B27, ISS-A01, ISS-A06, and ISS-A16 showed significant (*p* < 0.05) L5178Y-R cell growth inhibition, compared with an untreated control. The rhizosphere *Micromonospora echinospora* isolate ISS-A16 showed the highest (90.48%) percentage of lymphoma cells growth inhibition and SI (19.1) for PBMC, whereas the *Bacillus subtilis* ISE-B26 isolate caused significant (*p* < 0.01) growth inhibition (84.32%) and a SI of 5.2. Taken together, results of the present study evidenced antitumor effects by *I. sonorae* endophytic and rhizosphere bacteria culture extracts. Further research will involve the elucidation of the compounds that exert the antitumor activity and their evaluation in pre-clinical studies.

## 1. Introduction

Cancer is a multifactorial disease with a worldwide distribution and a current mortality rate of 9.9 million [1]. In particular, lymphoma has an incidence of 627,439 cases and a mortality rate of 283,169 cases per year, which represents one of the 10 most prevalent cancers globally [2]. Although chemotherapy is the treatment of choice, drug resistance has prompted an increasing demand for new antitumor compounds with low or null toxicity to normal cells [3,4].

Bioactive compounds produced by endophytic bacteria, isolated from medicinal plants, represent a source of new drugs with antitumor potential [5]. Medicinal plants have been extensively studied in recent years in the search for antitumor agents. However, the high production and processing costs of such compounds have been a limitation for their clinical use. This has prompted the isolation of endophytic and soil microorganisms to find new compounds with antitumor activity [6]. To date, a number of exopolysaccharides, aminoglycosides, oligosaccharides, anthracyclines, flavonoids, xioolysaccharides, and terpenes from rhizosphere endophytic microorganisms associated with medicinal plants have been identified [7,8,9].

Microorganisms and compounds with antitumor activity from plants of the Cucurbitaceae family have been identified [10,11,12]. *Ibervillea sonorae*, also known as “Wereke”, is a Cucurbitaceae plant, native to northern Mexico, that has been used in traditional medicine against diabetes, neurodegenerative diseases, and skin cancer [13]. Several studies on *I. sonorae* methanol extracts have demonstrated their in vitro activity against cervical cancer (HeLa), hepatocarcinoma (HepG2), lymphoma (L5178Y-R), and lung cancer (A549) [14,15,16]. However, the antitumor potential of endophytic and rhizosphere microorganisms associated with *I. sonorae* has not yet been reported. The aim of the present study was to isolate and evaluate the antitumor effect of methanol and ethyl acetate extracts from *I. sonorae* endophytic and rhizosphere microorganisms, in an in vitro murine L5178Y-R lymphoma model.

## 2. Materials and Methods

### 2.1. Plant Material

*I. sonorae* was collected in Huatabampo, Sonora (26°39′23″ N and 109°22′49″ W). The collected specimens were identified by M. González Álvarez and J. A. Verduzco Martínez, in the herbarium of the Facultad de Ciencias Biológicas at Universidad Autónoma de Nuevo León, México, with voucher number 025589.

### 2.2. Isolation of Endophytic and Rhizosphere Bacteria

Plant roots were subjected to a series of washings with soap and water to avoid contamination by epiphytes [17]. Internal cuts of 1 cm^2^ were then made, followed by 1 min washing in 1% NaClO, 1 min washing in sterile distilled water, 1 min treatment with 70% ethanol, two consecutive rinses with sterile distilled water for 1 min, and a washing with PBS solution (negative control), under sterile conditions in a biosafety level 2 laminar-flow hood [18]. Plant tissue was macerated in 9 mL of PBS with a sterile mortar, after which 100 µL were inoculated in triplicate on fluoride mannitol agar (AFM), casein starch agar (ACA), malt extract agar (ISP2), extract agar of yeast (AEL), and inorganic salt-starch agar (ISP4), supplemented with 50 µg/mL nalidixic acid (Nalixone) and 50 µg/mL cyclohexamide (Sigma-Aldrich, St. Louis, MO, USA), and incubated at 28 °C for two weeks [19]. 

For the isolation of rhizosphere bacteria, one gram of soil was dissolved in 9 mL of PBS. The tube was left in incubation for 15 min at 70 °C, after which 100 µL were inoculated in AFM, ACA, AEL, ISP2, and ISP4 agar, supplemented with 50 µg/mL nalidixic acid and 50 µg/mL cyclohexamide and incubated at 28 °C for three weeks [20,21]. Isolated colonies were preserved in GYM *Streptomyces* broth, supplemented with 30% glycerol at −70 °C, until use.

### 2.3. Characterization of Isolated Strains

Morphological characterization of isolated bacteria was performed according to their macroscopic growth characteristics, including shape, size, substrate color, and Gram stain. Bacteria were also identified by PCR, targeting the 16S ribosomal RNA (rRNA) gene [22]. Reactions were performed in a 20 μL volume, containing 100 ng of DNA, 10 μL of Ruby Taq Master 2X (Jena Bioscience, Jena, Germany), 0.5 μL of 27 F (5′-AGAGTTTGATCCTGGCTCAG-3′), 0.5 μL of 10 µM 1492 R (5′-TACGGYTACCTTGTTACGACTT-3′), and 8 µL of water. Conditions included a denaturation cycle of 95 °C for 5 min, 35 cycles of 94 °C for 30 s, 60 °C for 45 s, and 72 °C for 90 s, followed by an extension cycle at 72 °C for 8 min. PCR products (1600 bp) were purified by the agarose gel extraction kit (Jena Bioscience) and sequenced on an ABI PRISM 310 TM sequencer (PE Applied Biosystems, Foster City, CA, USA) at the DNA Synthesis and Sequencing Unit of the Biotechnology Institute of the Universidad Autónoma de México in Cuernavaca, Morelos. The nucleotide sequences obtained were analyzed, using the Ezbiocloud database of 16S based ID (https://www.ezbiocloud.net/) (Consulted on 17 December 2021) for the identification of the isolated bacteria.

Isolated strains were analyzed by MALDI-TOF MS (Microflex LT System, Bruker Daltonics, Bremen, Germany), using the MALDI Biotyper 3.0 software, in the Infectology Laboratory of University Hospital “Dr. José Eleuterio González” at Universidad Autónoma de Nuevo León, México. The analysis showed a score of 2.0 to 3.0 for a reliable identification at the species level, 1.7 to 1.9 for identification at the genus level, and a score of <1.7 was considered as an unreliable identification [23].

### 2.4. Extracts Preparation

Bacteria were cultured in 120 mL of GYM broth for four weeks, under shaking (120 rpm) at 28 °C. Cultures were then centrifuged at 3000 rpm for 10 min and biomass and supernatants were separated. For the preparation of methanol extracts, biomass was suspended (*w*/*v*) with two volumes of methanol, homogenized, and sonicated for five minutes, after which it was stirred for 48 h at 120 rpm at 25 °C in darkness [24]. To prepare ethyl acetate extracts, 50 mL of the supernatant was placed in a separatory funnel with 100 mL of the solvent, which was manually shaken for five minutes and the organic phase was discarded [25]. Solvents were removed with a SpeedVac SPD121P concentrator (Thermo Scientific, San Jose, CA, USA) at 35 °C. Then, 1 mg of solvent-based extracts was suspended in 20 µL of 99.5% dimethylsufoxide (DMSO, Sigma-Aldrich, St. Louis, MO, USA), filtered by 0.20 µm filters (Corning Incorporated, Corning, NY, USA), and stored at −20 °C, until use [26]. The final DMSO concentration in cell cultures was less than 1%, which did not affect cell viability. Extract yields were analyzed by the following Formula (1):

% Yield = (grams of dried extract/grams of biomass) (100)(1)

### 2.5. Tumor and Normal Cells

Murine L5178Y-R lymphoma cells (ATCC CRL-1722) and human peripheral blood mononuclear cells (PBMC) (obtained from 20 mL to 30 mL of blood from a healthy volunteer donor (three experiments were performed), using Ficoll-Paque PLUS (GE Healthcare Life Sciences, Pittsburgh, PA, USA)) were maintained in RPMI 1640 culture medium (Life Technologies, Inc., Grand Island, NY, USA), supplemented with 10% inactivated fetal bovine serum (Life Technologies, Inc., Grand Island, NY, USA), and 1% antibiotic/antimicotic solution (Life Technologies, Inc., Grand Island, NY, USA). This medium was referred as complete RPMI 1640 culture medium. Cells were cultured at 37 °C in an atmosphere of 5% CO_2_ in 95% air. 

### 2.6. Effect of Extracts on Tumor Cell Growth

L5178Y-R cell suspensions (100 μL) were cultured at a density of 1 × 10^4^ cells/well and PBMC at 1 × 10^5^ cells/well, into flat-bottomed 96-well plates (Corning Incorporated) in complete RPMI 1640 culture medium. After 24 h of incubation, cells were further incubated in triplicate for 48 h at 37 °C in 5% CO_2_ with 1:2 serial dilutions of 1 mg/mL stock extracts, resulting in concentrations ranging from 15.6 μg/mL to 500 μg/mL, in a final volume of 200 µL. Tumor cell growth was then evaluated by the colorimetric 3-[4,5-dimethylthiazol-2-yl]-2,5-diphenyltetrazoliumbromide (MTT; Affymetrix, Cleveland, OH, USA) reduction assay by adding 15 µL of MTT (0.5 mg/mL final concentration) and incubating at 37 °C for an additional 3 h. Formazan crystals were dissolved with DMSO and optical densities (OD) were measured at 570 nm in a MULTISKAN GO microplate reader (Thermo Fisher Scientific, Waltham, MA, USA). Cell growth inhibition percentage was calculated as follows: % Growth inhibition = 100 − ((OD_570_ in extract-treated cells/OD_570_ in untreated cells) (100)), using 0.05 µg/mL vincristine sulphate (VC; Hospira, Warwickshire, UK) as a positive control. Logarithmic scale concentrations were plotted against percent growth inhibition to determine IC_50_ values, which were used to determine the selectivity index (SI). This index was calculated by dividing the IC_50_ of normal cells by that of tumor cells [27].

### 2.7. Statistical Analysis

Percent growth inhibition results were expressed as mean ± SD of triplicate determinations from three independent experiments. Level of significance was evaluated by the Kruskal–Wallis test. Statistical analyses were performed using the Graph Pad Prism 7 program (GraphPad Software Inc., San Diego, CA, USA).

## 3. Results

### 3.1. Isolation of Endophytic and Rhizosphere Bacteria from I. sonorae

**Figure 1:**
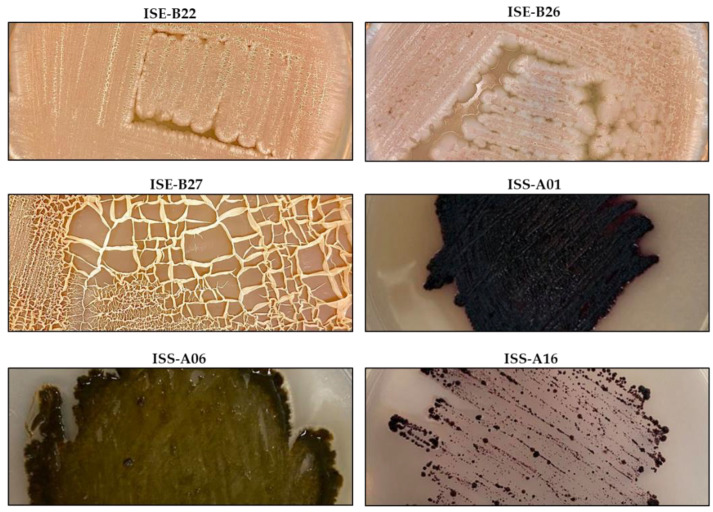
Isolated endophytic and rhizosphere Gram-positive bacteria from *Ibervillea sonorae*.

Morphologically, bacteria showed different growth forms in fluorine mannitol agar and ISP4 agar (BD Biosciences, San Jose, CA, USA) (Figure 1). Endophytic bacteria (ISE) generally showed irregular growth, white color, and wavy edges; whereas rhizosphere bacteria (ISS) showed a punctate, granular, wavy growth, and various color tones (Table 1). After 28 d of fermentation, methanol extract yields were from 1.4% to 3.57%, whereas ethyl acetate extracts had yields of 0.11% to 0.3%.

### 3.2. Effect of I. sonorae Endophytic and Rhizosphere Bacteria Extracts on L5178Y-R Lymphoma Cells Growth

**Figure 2:**
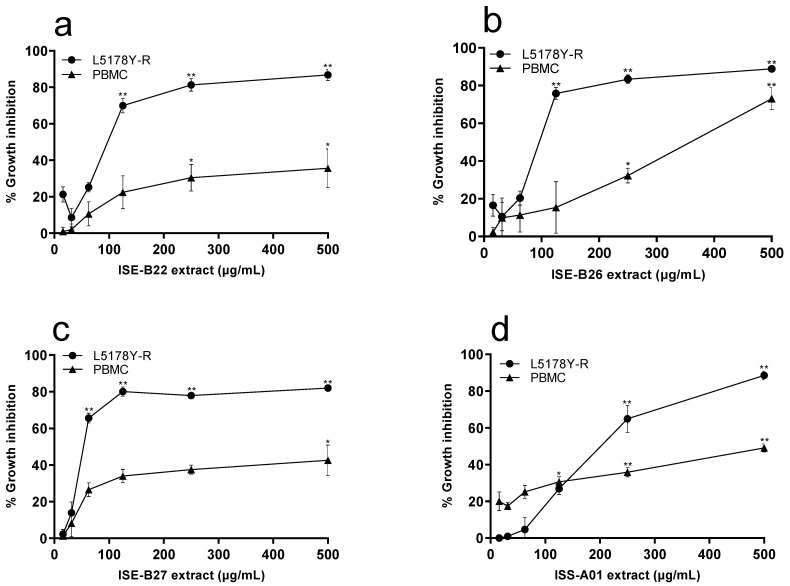

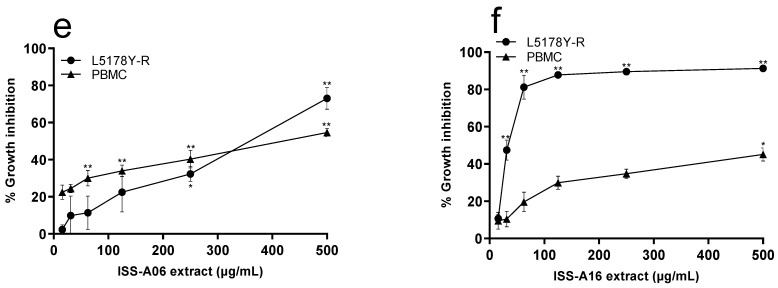
Tumor cell growth inhibition by methanol extracts from *I. sonorae* endophytic and rhizosphere bacteria cultures. L5178Y-R cells were placed at a concentration of 1 × 10^4^ cells/well and PBMC at 2 × 10^5^ cells/well, and treated with ethyl acetate bacterial extracts at concentrations ranging from 15.62 µg/mL to 500 µg/mL for 48 h. (**a**) ISE-B22, (**b**) ISE-B26, (**c**) ISE-B27, (**d**) ISS-A01, (**e**) ISS-A06, and (**f**) ISS-A16. Cell growth inhibition was assessed by the MTT reduction assay, measuring ODs at 570 nm. Vincristine (VC) was used as a positive control at a concentration of 0.05 µg/mL, as detailed in the text. VC caused 69.98% and 16% growth inhibition against L5178Y-R and PBMC, respectively. Data represent mean ± SD of triplicates from three independent experiments. * *p* < 0.05; ** *p* < 0.01, as compared with untreated control.

**Figure 3:**
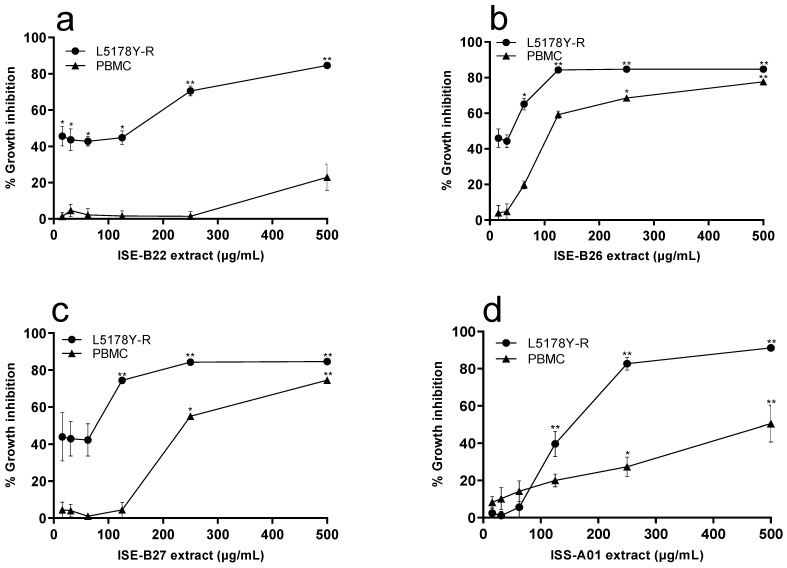

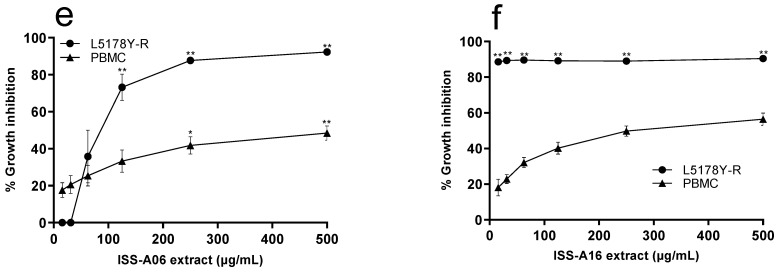
Tumor cell growth inhibition by ethyl acetate extracts from *I. sonorae* endophytic and rhizosphere bacteria cultures. L5178Y-R cells were placed at a concentration of 1 × 10^4^ cells/well and PBMC at 2 × 10^5^ cells/well, and treated with ethyl acetate bacterial extracts at concentrations ranging from 15.62 µg/mL to 500 µg/mL for 48 h. (**a**) ISE-B22, (**b**) ISE-B26, (**c**) ISE-B27, (**d**) ISS-A01, (**e**) ISS-A06, and (**f**) ISS-A16. Cell growth inhibition was assessed by the MTT reduction assay, measuring OD at 570 nm. Vincristine (VC) was used as a positive control at a concentration of 0.05 µg/mL, as detailed in the text. VC caused 69.98% and 16% growth inhibition against L5178Y-R and PBMC, respectively. Data represent mean ± SD of triplicates from three independent experiments. * *p* < 0.05; ** *p* < 0.01, as compared with untreated control.

We evaluated the effect of methanol and ethyl acetate extracts from isolated bacteria at concentrations ranging from 15.62 µg/mL to 500 µg/mL on L5178Y-R and human PBMC viability. We showed that methanol and ethyl acetate bacterial extracts inhibited L5178Y-R cells growth (Figure 2 and Figure 3). The endophyte ISE-B27 and the rhizosphere ISS-A16 strain methanol extracts caused the lowest IC_50_ at 60.03 μg/mL and 34.41 µg/mL, respectively, whereas the endophyte ISE-B26 and the rhizosphere isolate ISS-A16 ethyl acetate extracts induced the lowest IC_50_ at 26 µg/mL and 4.77 µg/mL, respectively (Figure 2). Ethyl acetate extracts from ISS-A16 and ISE-B26 bacteria presented the highest percentage of inhibition with 89.18% (*p* < 0.01) and 84.32% (*p* < 0.01), respectively, at 125 µg/mL (Figure 3). Based on the IC_50_, we observed SIs from 1.5 to 19.1 for the methanol extracts and from 4.1 to 14.5 for the ethyl acetate extracts, using PBMC as normal control cells (Table 2).

### 3.3. Molecular Identification and Mass Spectrometry of I. sonorae Endophytic and Rhizosphere Bacteria with Antitumor Activity

Identification of isolated microorganisms was performed by sequencing the 16S ribosomal gene and by mass spectrometry of those that inhibited L5178Y-R cells growth (Figure 1). The amplified region (16S) from isolated bacteria was sequenced and analyzed, using the Ezbiocloud database of 16S rRNA sequences (16S based ID). ISE-B22, ISE-B26, and ISE-B27 bacterial strains were identified as *Bacillus* sp. with 99%, 99.85%, and 100% identity, respectively, whereas ISS-A01, ISS-A06, and ISS-A16 bacterial strains were identified as *Micromonospora purpureochromogene*, *M. halophytica*, and *M. echinospora* with 99.78%, 99.68%, and 100% identity, respectively. Mass spectrophotometric analysis of ISE-B26 and ISE-B27 strains showed a relationship with *Bacillus subtilis* with 2.57 and 2.12 scores, respectively. The isolate ISE-B22 was identified as a co-culture (*Bacillus subtilis* and *Bacillus mojavensis*) with a score of 2.5 for each species of *Bacillus*. The ISS-A16 strain was identified as *M. echinospora* with a score of 1.80, whereas ISS-A01 and ISS-A06 strains were not identified, because they were not found in the MALDI–TOF MS system database (Table 3).

## 4. Discussion

Lymphoma continues to represent one of the 10 leading causes of cancer deaths worldwide [2]. Despite advances in cancer therapies, they continue to present a great disadvantage, involving drug resistance and toxicity to normal cells [28]. Therefore, the search for new compounds with higher selectivity, less invasiveness, and lower toxicity to normal cells has become a current research interest [5,27]. Various plant-derived compounds with antitumor activity, such as vincristine, vinblastine, taxol, doxorubicin, resveratrol, curcumin, betulinic acid, rutin, and cucurbitacins, have been identified. However, the isolation of new compounds from endophytic microorganisms, or from the rhizosphere, such as azalomycin, streptocarbazole, actinomycin, streptomyceamide, and neoantimycin, for easy, fast, and economic production has demonstrated the importance of these microorganisms as potential sources of antitumor agents [7,8,9,29,30,31,32].

In the present study, we isolated different strains of *Bacillus*, which relates to other reports, showing the presence of these bacteria in 50% of endophytes from cucurbitaceous plants [33]. We also isolated various species of *Micromonospora* from the rhizosphere, such as *M. echinospora*, *M. inositola,* and *M. terminaliae*, which are commonly found in marine sediments, probably due to the area of plant collection on the southern coast shores of the state of Sonora [34,35].

A number of endophytic and rhizosphere Gram-positive bacteria present in various environments have shown in vitro anticancer effects against tumor cell lines, such as liver cancer (Hep-G2), cervical cancer (HeLa), lung cancer (A549), breast cancer (MCF7), and myeloid leukemia (SK-MEL-28) [36,37] cells. These results are related to the inhibitory effect of methanol and ethyl acetate extracts from isolated *I. sonorae* microorganisms on L5178Y-R lymphoma cell growth, as shown in the present study (Figure 2 and Figure 3). Furthermore, ethyl acetate fractions of the *B. subtilis* strains ISE-B22, ISE-B26, and ISE-B27, showed an IC_50_ of 56.8 ± 1.143, 26 ± 1.404, and 39.74 ± 1.217 µg/mL, respectively (Table 2), which indicates a significant antitumor potential. In this regard, *B. subtilis* strains have shown antitumor activity against MCF7, HeLa, Hep G2, and CaCo2 tumor cell lines, probably mediated by compounds such as gallic acid, eicosan, pentacosane, amicoumacin, bacilosarcin e-Poly-L-Lysine, and surfactin [38,39,40,41,42,43,44].

In addition, extracts based on isolated *Micromonospora* species possessed higher antitumor activity than that of *Bacillus* species, which may be due to a higher amount of synthesized compounds [37]. In this regard, *Micromonospora* sp. isolated from marine sediment was shown to synthesize the cytotoxic compound IB-96212 against the lymphoma P388 and the myeloid leukemia SK-MEL-28 at concentrations lower than 1 µg/mL [45]. Some *Micromonospora* species isolated from rhizosphere and marine sediments have been shown to synthesize compounds of pharmacological application in oncological processes, including anthracyclines [46,47,48,49].

The search for new sources of compounds with antitumor potential represents a current priority in the pharmaceutical area due to the emergence of new variants that are resistant to conventional antineoplastic drugs. Microorganisms associated with medicinal plants (endophytes or rhizosphere) produce bioactive compounds of high selectivity for cancer cells, whose mechanisms are yet to be elucidated. 

## 5. Conclusions

The presence of Gram-positive bacteria associated with medicinal plants shows the biotechnological potential of sources of production of new molecules with antitumor activity. Methanol or ethyl acetate extracts of microorganisms associated with *I. sonorae* bioactive compounds demonstrated their antitumor potential in a murine model of lymphoma.

## Figures and Tables

**Table 1:** Colonies morphological characterization and yields of *I. sonorae* endophytic and rhizosphere bacteria extracts.

Strain	Color ^b^	Shape	Size ^a^	Methanol Extract Yield	Ethyl Acetate Extract Yield ^c^
ISE-B22	#FFD39B	Punctiform	S	1.67%	0.3%
ISE--B26	#CDAA7D	Filamentous	I	1.69%	0.12%
ISE-B27	#DEB887	Filamentous	L	3.57%	0.16%
ISS-A01	#551A8B	Granular	S	1.4%	0.19%
ISS-A06	#3D3D3D	Granular	I	1.7%	0.11%
ISS-A16	#A52A2A	Punctiform	I	2.27%	0.12%

^a^ Size (diameter in millimeters): S = small (<2 mm); I = intermediate (2 to 3 mm); L = large (>3 mm). ^b^ Colony colors were based on those from the web page http://www.webusable.com/coloursTable.htm (accessed on 22 October 2021). ^c^ Ethyl acetate yields were calculated per 50 mL of supernatant.

**Table 2:** SI and IC_50_ of isolated bacterial culture extracts on murine lymphoma L5178Y-R and PBMC growth.

Isolate	Ethyl Acetate IC_50_ (µg/mL)			Methanol IC_50_ (µg/mL)		
	L5178Y-R	PBMC	SI	L5178Y-R	PBMC	SI
ISE-B22	56.83 ± 1.143 ^a^	669.3 ± 0.127	11.8	94.7 ± 1.528	724 ± 1.116	7.6
ISE-B26	26 ± 1.404	136.1 ± 1.625	5.2	91.82 ± 1.564	325.3 ± 1.54	3.5
ISE-B27	39.74 ± 1.217	267 ± 1.768	6.7	60.03 ± 1.476	781.1 ± 0.892	13.0
ISS-A01	147 ± 1.929	596.8 ± 1.268	4.1	194.6 ± 1.996	715.1 ± 1.216	3.7
ISS-A06	91.25 ± 1.710	562 ± 1.227	6.2	325.3 ± 1.547	473.6 ± 1.265	1.5
ISS-A16	4.77 ± 1.60	276.5 ± 1.583	14.5	34.41 ± 1.742	655.9 ± 1.334	19.1

^a^ Values represent mean ± SD of three independent experiments.

**Table 3:** Molecular identification based on the 16S ribosomal gene and mass spectrometry of endophytic and rhizosphere bacteria with antitumor activity.

Isolated	Ribosomal Gene 16S		MALDI-TOF MS		Classification
	EZbiocloud	Similarity	Identification	Score	
ISE-B22	*Bacillus* sp.	99%	*Bacillus subtilis*	2.5	*Bacillus* sp.
			*Bacillus mojavensis*		
ISE-B26	*Bacillus subtilis*	99.85%	*Bacillus subtilis*	2.57	*B. subtilis*
ISE-B27	*Bacillus subtilis*	100%	*Bacillus subtilis*	2.12	*B. subtilis*
ISS-A01	*Micromonospora purpureochromogenes*	99.78%	ND ^a^	ND	*M. purpureochromogenes*
ISS-A06	*Micromonospora halophytica*	99.68%	ND	ND	*M. halophytica*
ISS-A16	*Micromonospora echinospora*	100%	*M. echinospora*	1.8	*M. echinospora*

^a^ ND: Not determined.

## Data Availability

The datasets generated and/or analyzed during the present study are available from the corresponding author on reasonable request.

## References

[1] Sung H., Ferlay J., Siegel R.L., Laversanne M., Soerjomataram I., Jemal A., Bray F. (2021). Global Cancer Statistics 2020: GLOBOCAN estimates of incidence and mortality worldwide for 36 cancers in 185 countries. CA Cancer J. Clin..

[2] International Agency for Research on Cancer Section of Cancer Surveillance. http://gco.iarc.fr/.

[3] Wang L., Qin W., Huo Y.J., Li X., Shi Q., Rasko J.E.J., Janin A., Zhao W.L. (2020). Advances in targeted therapy for malignant lymphoma. Signal Transduct. Target. Ther..

[4] Bailly C., Thuru X., Quesnel B. (2020). Combined cytotoxic chemotherapy and immunotherapy of cancer: Modern times. NAR Cancer.

[5] Christina A., Christapher V., Bhore S. (2013). Endophytic bacteria as a source of novel antibiotics: An overview. Pharmacogn. Rev..

[6] Singh M., Kumar A., Singh R., Pandey K.D. (2017). Endophytic bacteria: A new source of bioactive compounds. 3 Biotech.

[7] Zheng L.P., Zou T., Ma Y.J., Wang J.W., Zhang Y.Q. (2016). Antioxidant and DNA damage protecting activity of exopolysaccharides from the endophytic bacterium *Bacillus cereus* SZ1. Molecules.

[8] Ghosh A., Sutradhar S., Baishya D. (2019). Delineating thermophilic xylanase from *Bacillus licheniformis* DM5 towards its potential application in xylooligosaccharides production. World J. Microbiol. Biotechnol..

[9] Nafie M.S., Awad N.M., Tag H.M., Abd El-Salam I.M., Diab M.K., El-Shatoury S.A. (2021). Micromonospora species from rarely-exploited Egyptian habitats: Chemical profile, antimicrobial, and antitumor activities through antioxidant property. Appl. Microbiol. Biotechnol..

[10] Kim H.J., Park J.H.Y., Kim J.K. (2014). Cucurbitacin-I, a natural cell-permeable triterpenoid isolated from Cucurbitaceae, exerts potent anticancer effect in colon cancer. Chem. Biol. Interact..

[11] Güneş H., Alper M., Çelikoğlu N. (2019). Anticancer effect of the fruit and seed extracts of *Momordica charantia* L. (Cucurbitaceae) on human cancer cell lines. Trop. J. Pharm. Res..

[12] Salehi B., Quispe C., Sharifi-Rad J., Giri L., Suyal R., Jugran A.K., Zucca P., Rescigno A., Peddio S., Bobiş O. (2021). Antioxidant potential of family Cucurbitaceae with special emphasis on *Cucurbita* genus: A key to alleviate oxidative stress-mediated disorders. Phytother. Res..

[13] Alarcon-Aguilar F.J., Calzada-Bermejo F., Hernandez-Galicia E., Ruiz-Angeles C., Roman-Ramos R. (2005). Acute and chronic hypoglycemic effect of *Ibervillea sonorae* root extracts-II. J. Ethnopharmacol..

[14] Torres-Moreno H., Marcotullio M.C., Velázquez C., Ianni F., Garibay-Escobar A., Robles-Zepeda R.E. (2020). Cucurbitacin IIb, a steroidal triterpene from *Ibervillea sonorae* induces antiproliferative and apoptotic effects on cervical and lung cancer cells. Steroids.

[15] Quintanilla-Licea R., Gomez-Flores R., Samaniego-Escamilla M.Á., Hernández-Martínez H.C., Tamez-Guerra P., Morado-Castillo R. (2016). Cytotoxic effect of methanol extracts and partitions of two Mexican desert plants against the murine lymphoma L5178Y-R. Am. J. Plant Sci..

[16] Torres-Moreno H., Marcotullio M.C., Velazquez C., Arenas-Luna V.M., Hernández-Gutiérrez S., Robles-Zepeda R.E. (2020). Cucurbitacin IIb from *Ibervillea sonorae* induces apoptosis and cell cycle arrest via STAT3 inhibition. Anti-Cancer Agents Med. Chem..

[17] Glassner H., Zchori-Fein E., Compant S., Sessitsch A., Katzir N., Portnoy V., Yaron S. (2015). Characterization of endophytic bacteria from cucurbit fruits with potential benefits to agriculture in melons (*Cucumis melo* L.). FEMS Microbiol. Ecol..

[18] Shan W., Zhou Y., Liu H., Yu X. (2018). Endophytic actinomycetes from tea plants (*Camellia sinensis*): Isolation, abundance, antimicrobial, and plant-growth-promoting activities. BioMed Res. Int..

[19] Wang D.S., Xue Q.H., Ma Y.Y., Wei X.L., Chen J., He F. (2014). Oligotrophy is helpful for the isolation of bioactive actinomycetes. Ind. J. Microbiol..

[20] Hayakawa M., Yoshida Y., Iimura Y. (2004). Selective isolation of bioactive soil actinomycetes belonging to the Streptomyces violaceusniger phenotypic cluster. J. Appl. Microbiol..

[21] Ayoubi H., Mouslim A., Moujabbir S., Amine S., Azougar I., Mouslim J., Menggad M. (2018). Isolation and phenotypic characterization of actinomycetes from Rabat neighborhood soil and their potential to produce bioactive compounds. Afr. J. Microbiol. Res..

[22] Weisburg W.G., Barns S.M., Pelletier D.A., Lane D.J. (1991). 16S ribosomal DNA amplification for phylogenetic study. J. Bacteriol..

[23] Sogawa K., Watanabe M., Sato K., Segawa S., Ishii C., Miyabe A., Murata S., Saito T., Nomura F. (2011). Use of the MALDI BioTyper system with MALDI-TOF mass spectrometry for rapid identification of microorganisms. Anal. Bioanal. Chem..

[24] Prihanto A.A., Firdaus M., Nurdiani R. (2011). Endophytic fungi isolated from mangrove (*Rhyzopora mucronata*) and its antibacterial activity on *Staphylococcus aureus* and *Escherichia coli*. J. Food Sci. Eng..

[25] Minarni I.M.A., Julistiono H., Bermawie N., Riyanti E.I., Hasim A.E.Z.H. (2017). Anticancer activity test of ethyl acetate extract of endophytic fungi isolated from soursop leaf (*Annona muricata* L.). Asian Pac. J. Trop. Med..

[26] Nguyen S.T., Nguyen H.T.-L., Truong K.D. (2020). Comparative cytotoxic effects of methanol, ethanol and DMSO on human cancer cell lines. Biomed. Res. Ther..

[27] Ramírez-Villalobos J.M., Romo-Sáenz C.I., Morán-Santibañez K.S., Tamez-Guerra P., Quintanilla-Licea R., Orozco-Flores A.A., Romero-Arguelles R., Tamez-Guerra R., Rodríguez-Padilla C., Gomez-Flores R. (2021). In vitro tumor cell growth inhibition induced by *Lophocereus marginatus* (Dc.) S. Arias and Terrazas endophytic fungi extracts. Int. J. Environ. Res. Public Health.

[28] Raguz S., Yagüe E. (2008). Resistance to chemotherapy: New treatments and novel insights into an old problem. Br. J. Cancer.

[29] Dehelean C.A., Marcovici I., Soica C., Mioc M., Coricovac D., Iurciuc S., Cretu O.M., Pinzaru I. (2021). Plant-derived anticancer compounds as new perspectives in drug discovery and alternative therapy. Molecules.

[30] Vidal-Gutiérrez M., Torres-Moreno H., Hernández-Gutiérrez S., Velazquez C., Robles-Zepeda R.E., Vilegas W. (2021). Antiproliferative activity of standardized phytopreparations from *Ibervillea sonorae* (S. Watson) Greene. Steroids.

[31] Law J.W.F., Law L.N.S., Letchumanan V., Tan L.T.H., Wong S.H., Chan K.G., Ab Mutalib N.S., Lee L.H. (2020). Anticancer drug discovery from microbial sources: The unique mangrove Streptomycetes. Molecules.

[32] Ek-Ramos M.J., Gomez-Flores R., Orozco-Flores A.A., Rodríguez-Padilla C., González-Ochoa G., Tamez-Guerra P. (2019). Bioactive products from plant-endophytic Gram-positive bacteria. Front. Microbiol..

[33] Khalaf E.M., Raizada M.N. (2016). Taxonomic and functional diversity of cultured seed associated microbes of the cucurbit family. BMC Microbiol..

[34] Shirokikh I.G., Shirokikh A.A. (2017). Biosynthetic potential of actinomycetes in brown forest soil on the eastern coast of the Aegean Sea. Eurasian Soil Sci..

[35] Shrestha B., Nath D.K., Maharjan A., Poudel A., Pradhan R.N., Aryal S. (2021). Isolation and characterization of potential antibiotic-producing actinomycetes from water and soil sediments of different regions of Nepal. Int. J. Microbiol..

[36] Ferdous U.T., Shishir M.A., Khan S.N., Hoq M.M. (2018). *Bacillus* spp.: Attractive sources of anti-cancer and anti-proliferative biomolecules. Microb. Bioact..

[37] Qi S., Gui M., Li H., Yu C., Li H., Zeng Z., Sun P. (2020). Secondary metabolites from marine micromonospora: Chemistry and bioactivities. Chem. Biodiver..

[38] Torres-Moreno H., Velázquez C.A., Garibay-Escobar A., Curini M., Marcotullio M.C., Robles-Zepeda R.E. (2015). Antiproliferative and apoptosis induction of cucurbitacin-type triterpenes from *Ibervillea sonorae*. Ind. Crops Prod..

[39] Sansinenea E., Ortiz A. (2011). Secondary metabolites of soil *Bacillus* spp.. Biotechnol. Lett..

[40] Ramasubburayan R., Sumathi S., Magi Bercy D., Immanuel G., Palavesam A. (2015). Antimicrobial, antioxidant and anticancer activities of mangrove associated bacterium *Bacillus subtilis* subsp. subtilis RG. Biocatal. Agric. Biotechnol..

[41] Szende B., Szokan G., Tyiha E., Pal K., Gaborjanyi R., Almas M., Khlafulla A. (2002). Antitumor effect of lysine-isopeptides. Cancer Cell Int..

[42] Miller K.I., Qing C., Sze D.M.Y., Roufogalis B.D., Neilan B.A. (2012). Culturable endophytes of medicinal plants and the genetic basis for their bioactivity. Microb. Ecol..

[43] Aboul-Ela H., Shreadah M. (2012). Isolation, cytotoxic activity and phylogenetic analysis of *Bacillus* sp. bacteria associated with the red sea sponge *Amphimedon ochracea*. Adv. Biosci. Biotechnol..

[44] Meena K.R., Sharma A., Kanwar S.S. (2017). Microbial lipopeptides and their medical applications. Ann. Pharmacol. Pharm..

[45] Matloub A.A., Gomaa E.Z., Hassan A.A., Elbatanony M.M., El-Senousy W.M. (2020). Comparative chemical and bioactivity studies of intra- and extracellular metabolites of endophytic bacteria, *Bacillus subtilis* NCIB 3610. Int. J. Peptide Res. Ther..

[46] Fernández-Chimeno R.I., Cañedo L., Espliego F., Grávalos D., De la Calle F., Fernández-Puentes J.L., Romero F. (2000). IB-96212, a novel cytotoxic macrolide produced by a marine *Micromonospora* I. taxonomy, fermentation, isolation and biological activities. J. Antibiot..

[47] Ramalingam V., Varunkumar K., Ravikumar V., Rajaram R. (2019). Production and structure elucidation of anticancer potential surfactin from marine actinomycete *Micromonospora marina*. Process Biochem..

[48] Sousa T.D.S., Jimenez P.C., Ferreira E.G., Silveira E.R., Braz-Filho R., Pessoa O.D.L., Costa-Lotufo L.V. (2012). Anthracyclinones from *Micromonospora* sp.. J. Nat. Prod..

[49] Furumai T., Igarashi Y., Higuchi H., Saito N., Oki T. (2002). Kosinostatin, a quinocycline antibiotic with antitumor activity from *Micromonospora* sp. TP-A0468. J. Antibiot..

